# Reactive Oxygen and Nitrogen Species (RONS) and Cytokines—Myokines Involved in Glucose Uptake and Insulin Resistance in Skeletal Muscle

**DOI:** 10.3390/cells11244008

**Published:** 2022-12-11

**Authors:** Paola Llanos, Jesus Palomero

**Affiliations:** 1 Instituto de Investigación en Ciencias Odontológicas, Facultad de Odontología, Universidad de Chile, Santiago 8380544, Chile; 2 Centro de Estudios en Ejercicio, Metabolismo y Cáncer, Facultad de Medicina, Universidad de Chile, Santiago 8380453, Chile; 3 Department of Physiology and Pharmacology, Faculty of Medicine, Campus Miguel de Unamuno, Universidad de Salamanca, Av. Alfonso X El Sabio, 37007 Salamanca, Spain; 4 Institute of Neurosciences of Castilla y León (INCyL), 37007 Salamanca, Spain; 5 Institute of Biomedical Research of Salamanca (IBSAL), 37007 Salamanca, Spain

**Keywords:** ROS, RNS, hydrogen peroxide, nitric oxide, myokines, cytokines, glucose transport, GLUT4, insulin resistance, skeletal muscle fibers

## Abstract

Insulin resistance onset in skeletal muscle is characterized by the impairment of insulin signaling, which reduces the internalization of glucose, known as glucose uptake, into the cell. Therefore, there is a deficit of intracellular glucose, which is the main source for energy production in the cell. This may compromise cellular viability and functions, leading to pathological dysfunction. Skeletal muscle fibers continuously generate reactive oxygen and nitrogen species (RONS). An excess of RONS produces oxidative distress, which may evoke cellular damage and dysfunction. However, a moderate level of RONS, which is called oxidative eustress, is critical to maintain, modulate and regulate cellular functions through reversible interactions between RONS and the components of cellular signaling pathways that control those functions, such as the facilitation of glucose uptake. The skeletal muscle releases peptides called myokines that may have endocrine and paracrine effects. Some myokines bind to specific receptors in skeletal muscle fibers and might interact with cellular signaling pathways, such as PI3K/Akt and AMPK, and facilitate glucose uptake. In addition, there are cytokines, which are peptides produced by non-skeletal muscle cells, that bind to receptors at the plasma membrane of skeletal muscle cells and interact with the cellular signaling pathways, facilitating glucose uptake. RONS, myokines and cytokines might be acting on the same signaling pathways that facilitate glucose uptake in skeletal muscle. However, the experimental studies are limited and scarce. The aim of this review is to highlight the current knowledge regarding the role of RONS, myokines and cytokines as potential signals that facilitate glucose uptake in skeletal muscle. In addition, we encourage researchers in the field to lead and undertake investigations to uncover the fundamentals of glucose uptake evoked by RONS, myokines, and cytokines.

## 1. Introduction

The skeletal muscle is the largest organ in the body that performs different functions, including those related to the movement of the body such as stability, equilibrium, and locomotion; vital functions such as breathing; and those associated with the maintenance of metabolic homeostasis, in which the generation and expenditure of energy and heat production are critical [1,2]. The adequate interplay of these functions leads to the maintenance of life in organisms. Glucose is essential in metabolism since it is one of the main substrates that produces ATP, the key molecule that transfers energy during chemical reactions in organisms. To produce ATP, glucose needs to be transported from the extracellular space into the cytosol of the cell. This process is called glucose uptake, and it is critical in skeletal muscle since it provides enough glucose to the cell to produce ATP and satisfy the high demand for energy of the skeletal muscle [3]. Glucose uptake in skeletal muscle tissue is a process mainly regulated by insulin, which is a hormone synthesized in the pancreas and released into the blood stream, where it is transported until it binds to specific insulin receptors that are anchored at the plasma membrane of skeletal muscle cells. Once insulin binds to its receptor located at the extracellular face of the plasma membrane, an intracellular pathway is activated which drives the translocation of the glucose transporter (GLUT) from the cytosol to the plasma membrane. This skeletal muscle transporter, GLUT4, then drives the internalization of glucose from the extracellular space to the cytosol [3]. Therefore, the intracellular disposal of glucose is guaranteed for ATP production. When glucose uptake is dysregulated or impaired, the intracellular consumption of glucose is compromised, and this affects to the cellular metabolism. Moreover, since glucose is not transported to the cytosol of skeletal muscle cells, its concentration in blood increases, which is known as hyperglycemia, and this overstimulates the pancreatic beta cells to release more insulin; in this instance of the impairment of glucose transport, the insulin is not capable of internalizing the excess glucose. Therefore, the excess of glucose and insulin combined with the impairment of the intracellular internalization of glucose, known as glucose uptake, becomes a vicious circle that characterizes metabolic syndrome. The symptoms at the early stages of this syndrome include hyperglycemia, hyperinsulinemia, and the impairment of glucose uptake in skeletal muscle cells, which are not sensitive to the insulin effect, or insulin resistant. This can eventually evolve into type 2 diabetes (T2D) and other negative consequences of this pathology. There are polyfactorial causes that lead to the onset of metabolic syndrome, including abdominal obesity, elevated blood pressure, elevated triglycerides, low high-density lipoprotein cholesterol, and the previously mentioned hyperglycemia [4]. In addition, other factors include decreased physical activity, genetic predisposition, chronic inflammation, free fatty acids, and mitochondrial dysfunction [4]. All these factors and causes of metabolic syndrome are associated or mediated by oxidative stress, i.e., an excess of RONS [5]. The concept of oxidative stress (nowadays called oxidative distress) will be discussed later in this paper. Predictions indicate that by the year 2050, one in three persons will be diagnosed with diabetes [6]. Hence, this is a prime issue in healthcare worldwide. 

Skeletal muscle is mainly composed of fibers, which are postmitotic multinuclear cells formed from the fusion of single cells, known as myoblasts [7,8]. Muscle fibers continuously generate reactive oxygen and nitrogen species (RONS), and the production of RONS may be augmented in different situations, such as during contractile activity, inflammation, regeneration, and the aging of skeletal muscle [9]. When RONS are generated in excess and the intracellular antioxidant systems are unable to neutralize these highly reactive molecules, the skeletal muscle is exposed to oxidative distress and the RONS react with cellular macromolecules, such as lipids, proteins, carbohydrates, and nucleic acids, producing irreversible changes in those molecules and compromising the viability, integrity, and function of the cells [10]. However, when the level of intracellular RONS is moderate or relatively low, i.e., oxidative eustress, some of that RONS may act as signaling molecules, mediators or second messengers, which, through reversible redox reactions with specific residues of macromolecules, may modulate and regulate cellular signaling pathways that drive different cellular processes [10]. Over the last two decades, researchers in the field of redox biology have claimed and pointed out the importance of investigation and improving our knowledge regarding the function of RONS participating as signaling molecules in different pathways and how they are involved in the modulation, regulation, and control of cellular functions. In addition to the general physiological control of glucose uptake in skeletal muscle led by insulin, it has been proposed that RONS may be involved in the regulation of glucose uptake in skeletal muscle. However, it is uncertain whether this process might be independent or dependent of the effect of insulin [11,12,13]. Consequently, there is a need to investigate the role of RONS in the glucose uptake process in skeletal muscle. This knowledge may help us to find new targets for the treatment of the impairment of glucose uptake and insulin resistance manifested in type 2 diabetes, obesity, and aging.

Skeletal muscle is considered a secretory organ and liberates different cytokines, peptides, proteins, or hormones known as myokines that may have endocrine and auto/paracrine effects [14]. Myokines, such as myostatin, irisin and IL-6, among others, induce changes in the muscle itself, as well as in other organs and tissues [15]. These peptides, proteins or hormones regulate or alter the functionality and adaptation of muscle tissue, modifying its metabolism and other functions including hypertrophy or muscular atrophy, angiogenesis, and inflammatory processes [16]. Myokines may interfere with the prevention of obesity, metabolic syndrome, and type 2 diabetes. Consequently, there is an important focus in biomedical research to evaluate the potential of myokines as new therapeutic targets [15]. It appears that either the auto/paracrine effect of different myokines or the endocrine effect of different hormones, cytokines and factors that interact with skeletal muscle may affect glucose uptake and insulin resistance. However, the molecular signaling pathways and the physiological impact of these molecules remain largely unexplored.

In our opinion, there is scarce and dispersed published information regarding the interaction between RONS and myokines, cytokines and hormones in the context of glucose uptake and insulin resistance in skeletal muscle. It appears that RONS, through redox signaling reactions, might affect the pathways controlled by myokines, cytokines, hormones and other factors which affect the direct or indirect uptake of glucose in skeletal muscle. On the other hand, myokines, cytokines and other factors might affect RONS generation, and this might have consequences for glucose uptake and insulin resistance in skeletal muscle. Therefore, the current knowledge in the field is uncertain.

The aim of this review is to disclose the current information regarding the interaction between RONS and myokines, cytokines, hormones, and other factors in the context of glucose uptake and insulin resistance in skeletal muscle. Furthermore, this review aims to highlight and identify the potential targets which might be used as interventions to improve glucose uptake and prevent insulin resistance in skeletal muscle. We underline the necessity of investigating redox-mediated processes associated with the mechanisms that control glucose uptake and, consequently, this could help prevent insulin resistance in skeletal muscle.

## 2. Reactive Oxygen and Nitrogen Species in Skeletal Muscle

Around 1954, the first report was published that indicated that skeletal muscle produces free radical intermediates [17]. Twenty years later, two studies indicated that exercise was related to the generation of products from different reactions that occur between free radicals, referred to today as RONS, and cellular biomolecules [18,19]. These findings were consolidated in the 1980s when it was demonstrated that intense exercise produces free radicals in skeletal muscle [20]. Studies in the 1990s uncovered the fact that the contractile activity of skeletal muscle produced specific reactive species. Thus, it was demonstrated that superoxide was released from the diaphragm when this muscle contracted [21], nitric oxide was released from skeletal muscle [22], and hydroxyl radicals were generated in skeletal muscle during contractions [23]. The previous studies and other derived investigations have built upon this knowledge and created the current scenario that describes the generation of reactive oxygen and nitrogen species in skeletal muscle. For more detail, the reviews of [9,24,25,26] may be consulted. 

Basically, the main ROS generated in skeletal muscle cells (and cells from other tissues) are free radical species, such as superoxide anion (O_2_·−) and hydroxyl radical (HO·), and others considered nonradical species, such as hydrogen peroxide (H_2_O_2_). All of these are considered reactive oxygen species (ROS) since they originate from a reduction of oxygen and the reactive atom is oxygen. In the case of reactive nitrogen species (RNS), which are reactive species generated from nitrogen, the most relevant are nitric oxide (NO·), which is a free radical species, and peroxynitrite anion (ONOO-), which is a nonradical species, although very reactive [25]. Superoxide and nitric oxide are the primary species generated in skeletal muscle fibers and lead to the formation of other secondary species such as hydrogen peroxide, hydroxyl radical and peroxynitrite, all of which may be generated in the intracellular and extracellular space, in the mitochondrial matrix, and in the mitochondrial intermembrane space. The contractile activity of skeletal muscle fibers evokes an increase in the intracellular generation of superoxide, hydrogen peroxide and nitric oxide [27,28,29,30]. In addition, contractile activity increases the production of superoxide, hydrogen peroxide, hydroxyl radicals and nitric oxide extracellularly in the muscle interstitial space [31,32]. Most of the studies indicated that the increase in ROS generation caused by contractions in skeletal muscle was attributed to the increase in mitochondrial respiration due to the high demand for energy production in the mitochondrial matrix by the electron transport chain and ATP synthase, which conduct the formation of ATP, which is the source of energy for contractile activity. Therefore, mitochondria were attributed as the main source of ROS production in skeletal muscle. However, later studies indicated a lower increase in ROS and discovered other nonmitochondrial sources for ROS generation [25]. Thus, NADPH oxidases were identified in the skeletal muscle plasma membrane [33], the sarcoplasmic reticulum [34], and T-tubules [35]. The product of NADPH oxidase activity is superoxide, and we know from the later studies that NADPH oxidases contribute substantially to the production of superoxide and, potentially, hydrogen peroxide in skeletal muscle [36]. There are other sources of superoxide production that are less studied that may contribute to intracellular superoxide production in skeletal muscle, such as the enzyme phospholipase A2 [37]. In addition, the extracellular release of superoxide driven by a lipoxygenase was identified in skeletal muscle [38]. Another enzyme that may contribute to superoxide generation in the extracellular space of skeletal muscle is the xanthine oxidase located in the endothelial cells associated with skeletal muscle. It has been reported that this enzyme increases the superoxide concentration in the extracellular fluid of skeletal muscle during contractile activity [39].

Nitric oxide, the primary reactive nitrogen species (RNS), is generated enzymatically through nitric oxide synthase (NOS). NOS catalyzes the conversion of the amino acid L-arginine into L-citrulline, with cofactors NADPH and oxygen, and generates nitric oxide [25,26]. There are three NOS isoenzymes: neuronal (nNOS), endothelial (eNOS) and inducible (iNOS). nNOS is expressed in neurons and skeletal muscle, eNOS is expressed mainly in endothelial cells and in skeletal muscle with less expression, and iNOS appears to be associated with inflammatory processes that, in some circumstances, might affect the skeletal muscle [29]. Balon and Nadler were the first to identify the release of NO from skeletal muscle, which was augmented by contractile activity [22]. Other studies reported that skeletal muscle releases nitric oxide to the extracellular space, although they were uncertain which cells (i.e., skeletal muscle fibers, lymphocytes, fibroblasts, and endothelial cells) originate NO and the contribution they make to the total extracellular NO concentration [40,41]. Further work uncovered the release of NO specifically from skeletal muscle cells, such as myotubes [27,32]. In addition, it was indicated that the contractive activity of skeletal muscle produced an increase in the intracellular concentration of NO in a model of myotubes of a rat skeletal muscle origin [27]. Further studies, using the model of isolated single muscle fibers, demonstrated that contractile activity induced in fibers by electrical stimulation produced a rapid increase in the intracellular concentration of NO, which also rapidly decreased when contractile activity ceased [29]. Moreover, studies performed with a model of skeletal muscle fibers subjected to a protocol of passive elongation showed that the passive stretch applied to fibers isolated from old mice produced an increase in nitric oxide intracellular activity [42]. 

Hydrogen peroxide, hydroxyl radical and peroxynitrite are reactive species derived from the primary RONS, superoxide and nitric oxide, that play a relevant role in oxidative distress [43]. In skeletal muscle cells and other tissues, hydrogen peroxide is enzymatically generated in the cytosol, the mitochondrial matrix, and the extracellular space. Hydrogen peroxide is generated by the dismutation of superoxide in a reaction that is catalyzed by the enzyme superoxide dismutase (SOD). There are SOD isoenzymes that are expressed in different subcellular compartments. Manganese-SOD (Mn-SOD) is expressed in the mitochondrial matrix, copper/zinc-SOD (Cu/Zn-SOD) is expressed in the cytosol and mitochondrial intermembrane space, and there is an extracellular SOD (ec-SOD) that appears in the interstitial fluid in skeletal muscle [25,26]. Therefore, hydrogen peroxide is generated at different subcellular locations. When H_2_O_2_ concentration is high, it may appear oxidative distress in the cell. However, a lower H_2_O_2_ concentration leads to oxidative eustress, which is crucial for regulating and maintaining cellular processes through the modulation of different signaling pathways in the cell [10,43]. H_2_O_2_ may produce another secondary reactive species, such as hydroxyl radicals (HO·). This may occur when H_2_O_2_ is in the presence of divalent metals, mainly iron (Fe^++^), and H_2_O_2_ is decomposed to HO· by the Fenton reaction. The hydroxyl radical is a strong oxidant that is not diffusible through membranes and reacts with every close molecule. This produces alterations in macromolecules (lipids, proteins, and nucleic acids), which may disturb cellular homeostasis and other functions [25,26]. In other words, the hydroxyl radical mainly evokes oxidative distress. Peroxynitrite (ONOO-) is produced in a reaction between nitric oxide and superoxide [44]. This reaction is three times faster compared with the reaction of superoxide dismutation that produces hydrogen peroxide. Therefore, this is the primary reaction when both superoxide and hydrogen peroxide appear close together. Peroxynitrite shows a high oxidizing power and is very reactive; moreover, it may oxidize thiol groups of peptides, damage DNA and cause the nitration of proteins [25,26]. Hence, an abundance of peroxynitrite may potentially evoke oxidative distress.

Concomitant to RONS generation, cells are equipped with an antioxidant system that neutralizes the activity of reactive molecules. Therefore, there is a balance between the production and elimination of RONS to maintain the level of RONS at a steady state, facilitating homeostasis and cellular function. A moderate increase in the level of ROS is critical to trigger and modulate cellular signaling pathways that control several cellular functions. As mentioned above, this state is referred to as oxidative eustress [10]. However, when the level of RONS is elevated, if there is an excess of RONS being produced or the antioxidant system is unable to neutralize the RONS produced, then these reactive species interact with and oxidize other molecules (lipids, proteins, DNA, and carbohydrates), causing oxidative damage to them and possibly affecting their normal functions, which, ultimately, could compromise cellular homeostasis and viability. This state, already mentioned above, is currently referred to as oxidative distress in redox biology [10], in place of the classical term of oxidative stress.

The cellular antioxidant system is constituted of both an enzymatic and nonenzymatic system. The main antioxidant enzymes are superoxide dismutase (SOD), which catalyzes the conversion of superoxide anions into hydrogen peroxide; glutathione peroxidase (GPx), which neutralizes hydrogen peroxide to convert it to water and appears at different subcellular locations, particularly the mitochondria and cytosol; and catalase (CAT), which transforms hydrogen peroxide to water. In addition, there are accessory proteins that protect cells against oxidation, such as peroxiredoxin (PRX), glutaredoxin (GRX), and thioredoxin reductase (TRX). For more details, other comprehensive reviews in the field, i.e., [25], may be consulted. The nonenzymatic antioxidant system of the cell involves compounds such as glutathione, vitamins C and E, carotenoids, polyphenols, alpha-lipoic acid, coenzyme Q10, uric acid and bilirubin. These compounds are involved in thiol–disulfide exchange reactions and play a role in maintaining the redox balance in the cell [9,25]. Glutathione (GSH) is the most abundant nonenzymatic antioxidant in the cell, and it plays an important role due to its high reductive power. Glutathione reacts with different RONS, transferring protons and neutralizing these reactive species. Furthermore, glutathione is the substrate for the antioxidant enzyme GPx, and participates in the recycling of other antioxidant enzymes, such as TRX and GRX. Moreover, glutathione leads to the reduction of other antioxidants such as vitamins E and C [25]. Glutathione is critical in contractile activity and in the adaptation of skeletal muscle to exercise. This has been an important matter of investigation in the field of redox biology. It is out of the scope of this review, but readers may refer to other comprehensive key publications that describe the crucial role of glutathione in the redox biology of skeletal muscle. i.e., [45,46,47].

To recapitulate, RONS are generated in skeletal muscle at rest and during contractile activity. A high level of RONS might cause the excessive oxidation of macromolecules, which leads to cellular damage and dysfunction. However, a moderate RONS level is essential to modulate redox signaling processes that regulate cellular homeostasis and adaptation. The term RONS refers to several specific species. Some of them are mainly prone to induce oxidative distress, such as hydroxyl radicals and peroxynitrite. However, other RONS appear to be responsible for oxidative eustress and, currently, they are considered to be signaling molecules. These include nitric oxide and hydrogen peroxide, and even superoxide, which is the precursor to hydrogen peroxide. Over the last two decades, the focus of research in this area has been centered on nitric oxide, in addition to other important roles of NO in physiology and pharmacology, and especially on hydrogen peroxide. Different studies indicate that hydrogen peroxide has a crucial role in redox signaling pathways that regulate cellular homeostasis and other functions. Consequently, there is great interest in the field of redox biology and the knowledge continues to grow, although there is still much to discover. 

For a better understanding of the production of RONS and antioxidant systems in skeletal muscle cells, we recommend consulting some excellent illustrative figures published in key reviews in the field [25,26,48].

## 3. Glucose Uptake and Insulin Resistance in Skeletal Muscle 

Skeletal muscle tissue is critical for maintaining glucose due to its mass and high rate of metabolism, and thus, it is an important tissue in the context of impaired glucose homeostasis [49]. It is well known that insulin stimulates glucose uptake to extract glucose from the blood for storage through its conversion into glycogen [50]. Muscle glycogen is an important fuel source during exercise, and its depletion affects endurance exercise performance negatively [51]. Insulin regulates many biological functions in skeletal muscle and stimulates skeletal muscle glucose uptake, which is one of the most important processes regulated by insulin [52]. Insulin stimulates skeletal muscle glucose uptake through an increase in the GLUT4 translocation from intracellular storage vesicles to the sarcolemma and transverse tubules [53,54]. Insulin signaling is initiated through the binding and activation of its cell-surface receptor, which triggers a cascade of phosphorylation and dephosphorylation and the formation of second messengers, such as PIP3, activating the PI3 kinase and the subsequent phosphorylation of the Akt protein node [55]. After its activation, one of the targets of Akt is the Akt substrate of 160 kDa (AS160) [56]. The AS160 protein has a GTPase-activating domain which promotes GLUT4 translocation from GLUT4 storage vesicles (GSVs) to the plasma membrane (PM) to enhance glucose uptake [56]. Early studies demonstrated that acute insulin injection into the hindlimb muscle of rats resulted in a surge of GSVs to the PM fractions, accompanied by a decrease in intracellular GSVs [57]. In addition, other studies have elucidated the steps in the signaling cascades that involve the remodeling of the cytoskeleton, a process that underpins the mechanical movement of GLUT4 vesicles [58]. Glucose uptake is altered during insulin resistance, which is manifested by a decrease in insulin-dependent glucose uptake and which results from impaired insulin signaling and multiple post-receptor intracellular defects including impaired glucose transport, glucose phosphorylation and reduced glucose oxidation and glycogen synthesis [59,60].

The effect of the contractile activity of skeletal muscle on glucose uptake in this organ has been an issue of investigation. It has been demonstrated that insulin and contractile activity facilitate the glucose uptake in skeletal muscle by increasing the GLUT4 translocation to the cell plasma membrane. However, the signaling mechanism(s) that activate GLUT4 translocation and glucose uptake have not been fully elucidated [11]. There are studies that indicate that glucose uptake in skeletal muscle during dynamic exercise is up to 50 times higher compared to basal glucose uptake, and it is regulated through three important steps: glucose availability in muscle fiber, membrane transport, and flux through intracellular metabolism [13,61]. The mechanism through which contractile activity or exercise stimulates GLUT4 translocation and glucose uptake in skeletal muscle appears to be evoked from local factors, such as ROS, nitric oxide, calcium (Ca^++^), protein-dependent calmodulin kinase (CaMK) and AMP-activated protein kinase (AMPK) [13]. The pathways and mechanisms by which contractile activity increases glucose uptake within the muscle have not been fully elucidated, but there are studies that suggest that cell signaling pathways modulated by ROS and RNS are involved in these processes [13,62]. Glucose uptake in isolated skeletal muscle is associated with the production of hydrogen peroxide, which stimulates glucose transport [62]. This signaling mechanism is likely to be only partially overlapping with the insulin-induced glucose uptake mechanism [63]. Moreover, it has been reported that when nitric oxide production increases in skeletal muscle, due to the increase in the activity of nitric oxide synthase evoked by contractile activity or due to nitric oxide generation from nitric oxide donors, there is an enhancement of glucose uptake in this organ that is independent of the glucose uptake induced by insulin [64]. Thus, experimental evidence suggests that ROS, especially hydrogen peroxide, and RNS, particularly nitric oxide, play a role in the regulation of glucose transport during exercise and contractile activity. However, further investigations are required to establish the role of ROS and RNS in the signaling of glucose transport in skeletal muscle and to identify the mechanisms involved. It is important to uncover interactions between different RONS and other molecules associated with signaling pathways since these may be modulating glucose transport in skeletal muscle as well. The elucidation of these processes will allow us to know: (i) whether RONS are involved in the normal physiology of the insulin signaling cascade and glucose transport; (ii) if RONS contribute to the development of pathological states such as insulin resistance; and (iii) whether the elements of this system might emerge as pharmacological targets for the design of therapeutic interventions [65,66]. Although the underlying mechanisms causing diabetes have yet to be elucidated, skeletal muscle insulin resistance is a hallmark of diabetes and has been attributed to changes in reactive oxygen species activity and inflammation, among others [67]. 

In the context of RONS activity, it might be considered that an excess of RONS (oxidative distress) interacts with insulin signaling, and this might be what reduces glucose uptake [68]. However, the increase in RONS up to moderate physiological levels may improve insulin signaling and facilitate glucose uptake in skeletal muscle [68]. This observation could be translated to promising therapies to treat insulin resistance that appears in pathologies, such as diabetes, and physiological states, such as aging.

## 4. Effect of Cytokines on Skeletal Muscle 

Cytokines are peptides or hormones secreted by cells that have a specific effect on the interactions and communications between cells, including skeletal muscle cells [69,70]. A growing body of evidence has suggested that cytokines contribute substantially to the development of abnormal glucose homeostasis. However, others have shown their beneficial effects on glucose uptake and GLUT4 translocation in tissue [70] (Table 1).

Adiponectin is a 30 kDa, adipose-derived hormone secreted from adipose tissue by mature adipocytes [98]. High adiponectin levels are associated with a markedly reduced relative risk for T2D [99]. In addition, adiponectin regulates metabolism through blood glucose control and fatty acid oxidation, which is partly mediated by the downstream effects of adiponectin signaling in the skeletal muscle [100]. Treatment with adiponectin in rodents has been shown to decrease resting blood glucose levels and protect animals with diet-induced obesity from developing insulin resistance [101]. Preincubating isolated skeletal muscle or C2C12 myocytes with adiponectin stimulates glucose transport [71,72]. It has been reported that adiponectin increases glucose uptake in rat skeletal muscle cells via GLUT4 translocation and, subsequently, reduces the rate of glycogen synthesis and shifts glucose metabolism toward lactate production via the increased phosphorylation of AMP kinase and acetyl-CoA carboxylase, as well as through the oxidation of fatty acids [73]. Similarly, a role of regulating fatty acid and glucose metabolism in human skeletal muscle has also been reported [102].

Leptin is an adipocyte hormone that regulates energy homeostasis, which is mediated by the brain connection, and causes an increase in energy expenditure in animals [103]. The subcutaneous administration of leptin normalizes fasting plasma glucose in obese T2D rats [104]. In addition, this hormone enhances insulin sensitivity in the peripheral tissues, which is mediated by the central activation of the PI3K/Akt pathway [105]. Interestingly, it has been reported that leptin promotes glucose uptake in skeletal muscle via β2-adrenergic receptors, but not by AMPK [74]. Additionally, leptin enhances the intracellular GLUT4 transport in the skeletal muscle of *ob/ob* animals by reducing the expression and activity of the negative regulators of AS160 [75]. 

Monocyte chemotactic protein 1 (MCP-1) is a chemokine that regulates the recruitment, migration, and infiltration of monocytes/macrophages into tissue [106]. Elevated MCP-1 induces adipocyte dedifferentiation and contributes to pathologies associated with hyperinsulinemia and obesity, including T2D [107]. MCP-1 has been associated with skeletal muscle inflammatory markers during insulin resistance, as it was found that the muscle-specific overexpression of MCP-1 in transgenic mice induced the local recruitment of macrophages and altered local insulin sensitivity [76]. In addition, human skeletal muscle cells are highly sensitive toward MCP-1, which impairs insulin signaling and glucose uptake at concentrations even below those found in the circulation [77].

Interleukin-1β (IL-1β) is a potent proinflammatory cytokine that is crucial for host-defense responses to infection and injury [108]. Interestingly, C2C12 myotubes release IL-1β after the incubation with free fatty acids [109]. Recently, we reported that the activation of the NLRP3 inflammasome increases the IL-1β level and decreases GLUT4 translocation in adult skeletal muscle fibers in obesity-mediated insulin-resistant mice [67,110]. In accordance with this finding, high extracellular ATP levels, released through PANX1 channels, lead to the enhancement of the inflammatory state of IL-1β and to insulin resistance in the skeletal muscle fibers of obese mice [111]. Taken together, these findings suggest that muscle fibers may release IL-1β, which may disrupt glucose homeostasis due to insulin signaling alteration [110]. It is worthy of note that an increase in IL-1β in human skeletal muscle after exercise has been reported previously [112]; however, a compensatory effect is generated after sessions of exercise, decreasing the IL-1β level [113]. In addition, IL-1β impairs glucose uptake evoked by insulin and reduces the expression of IRS-1 in C2C12 cells [78].

Tumor necrosis factor-alpha (TNF-α) is an inflammatory cytokine produced by macrophages/monocytes during acute inflammation and is responsible for pleiotropic events within cells, leading to cellular death [114]. TNF-α signals, via interactions with neuronal-type nitric oxide synthase (nNOS), decrease specific forces in the skeletal muscle produced by contractions, and the reactive oxygen species generated in skeletal muscle mediate this response through NF-κB [115,116]. In humans, the circulating concentration of TNF-α is elevated in T2D, and this alteration is associated with impaired glucose tolerance and enhanced insulin resistance, in addition to an increased risk of T2D [117]. The excessive concentration of TNF-α negatively regulates insulin signaling in skeletal muscle and whole-body glucose uptake in humans via the inhibition of AS160 phosphorylation [79]. In addition, TNF-α produces insulin resistance in skeletal muscle through the activation of κB kinase inhibitors in a p38-MAPK-dependent manner, which produces serine phosphorylation in the insulin receptor and IRS-1, and thus prevents insulin from inducing tyrosine phosphorylation and impairs the corresponding activation of the PI3 kinase and Akt [80].

## 5. Myokines Involved in Glucose Uptake in Skeletal Muscle

Skeletal muscle is receiving increasing attention as an endocrine organ due to its release of peptides or proteins, referred to as myokines, that may influence the metabolism of virtually every organ in the body, including the muscle itself. Myokines present a new paradigm for understanding how muscles communicate with other organs [118,119]. However, special attention has also been given to the auto/paracrine effects of myokines within skeletal muscle affecting muscle functions [120]. This view, originally addressed over three decades ago, suggests that proteins and other peptides produced, expressed, and released by muscle fibers after exercise exert autocrine, paracrine, or endocrine effects through actions on their receptors [121]. However, a myokine may be secreted independently of muscle contractions [122]. It is worthy of note that myokines may represent potential therapeutic targets to combat obesity and associated metabolic disorders such as insulin resistance and T2D. Myokines produced by muscles during contraction may improve insulin sensitivity and glucose oxidation via autocrine actions [86]. A secretome-based analysis of the human myocyte culture medium has revealed more than 600 myokines to date [123]. However, most of these myokines are still insufficiently characterized. In addition, the signaling pathways of certain myokines are altered in the skeletal muscle of patients with T2D [124]. Taken together, this suggests that myokine secretion is an important factor contributing to the development of muscle metabolic defects in T2D. However, whether these myokines and their effects influence both glucose uptake and GLUT4 translocation remains largely unexplored. Thus, we present an update on the myokines that have been identified in association with glucose uptake and insulin resistance (Table 1).

Fibroblast growth factor 21 (FGF21) has emerged as a promising therapeutic agent for the treatment of obesity and T2D [125]. FGF21 is a protein preferentially expressed in the liver, but it has also been described as a myokine since its expression and secretion are regulated by insulin and Akt activation [126]. This peptide hormone is secreted by several organs and can act on multiple tissues to regulate energy homeostasis [127,128]. FGF21 has been proposed as a novel metabolic regulator given its ability to normalize glucose and lipid metabolism and prevent the development of obesity and diabetes [125]. Recently, it has been reported that FGF21 regulates glucose uptake through a mechanism mediated by GLUT4 and that is dependent on atypical PKC-ζ- in skeletal muscle [81]. FGF21 gene therapy in animals receiving a long-term high-fat-diet feeding or in *ob/ob* mice showed marked reductions in body weight, adipose tissue hypertrophy and inflammation, hepatic steatosis inflammation and fibrosis, and insulin resistance due to the higher expression of FGF21 [129]. 

Irisin is a myokine that is secreted after exercise and is associated with increased energy expenditure because of its ability to stimulate the browning of white adipose tissue [130]. In skeletal muscle, it has been proposed that irisin stimulates glucose uptake after the activation of AMPK in L6 myotubes [82]. Decreased irisin secretion contributes to muscle insulin resistance, which was observed in high-fat-diet mice when the insulin action was significantly inhibited [83,131]. In addition, irisin reverses insulin resistance in C2C12 muscle cells via the p38-MAPK-PGC-1α pathway and enhances mitochondrial function [84]. Irisin improves fatty acid oxidation and glucose utilization in T2D by regulating the AMPK signaling pathway [85]. 

The identification of IL-6 as a myokine has created much interest around its acting as a metabolic regulator molecule [132]. However, the elevation of systemic IL-6, often in obesity and metabolic syndrome, and the role of IL-6 in metabolic disease remains controversial [132]. After exercise, IL-6 plasma levels rise because of the increased local production in muscle, and this increase may enhance substrate metabolism and whole-body glucose homeostasis [133,134,135]. Acute IL-6 exposure increases glucose metabolism in resting human skeletal muscle without changing insulin-stimulated glucose transport and insulin signaling [87]. On the contrary, it has been reported that IL-6 administration increases insulin sensitivity in vitro in muscle via the AMP-activated protein kinase [86]. Interestingly, IL-6 induces lipolysis and free fatty acid release from adipocytes and skeletal muscle [136]. In addition, low-grade systemic inflammation is one of the earliest and main pathological events that might lead to the development of insulin resistance [137]. Therefore, IL-6 may exert both pro- and anti-inflammatory effects and may even promote muscle anabolism or catabolism depending on the target structure, the predominant cytokine environment, and the mode of release [138]. 

Apelin is a peptide secreted from various tissues that has been classically characterized as an adipokine. Moreover, it has been described as a myokine that improves glucose metabolism and shows antidiabetic properties [139,140]. Apelin knockout mice are insulin resistant, a condition that can be reversed after apelin treatment [88]. It has been reported that both short- and long-term apelin treatments improve insulin sensitivity in obese and insulin-resistant mice, mainly due to the increase in glucose uptake in skeletal muscle that was observed in [88,89]. Using in vivo and in vitro pharmacological and genetic approaches, the involvement of eNOS, AMP-activated protein kinase, and Akt was reported in apelin-stimulated glucose uptake in the soleus muscle [89]. In C2C12 myotubes, apelin increased glucose uptake and Akt phosphorylation [88]. Apelin expression is induced by exercise signaling pathways and secreted in vitro in human primary myotubes [141]. However, it has recently been reported that exercise-induced insulin sensitization occurs independently of plasma apelin changes [142]. Interestingly, apelin treatment increases complete fatty acid oxidation, mitochondrial oxidative capacity, and biogenesis in the muscle of insulin-resistant mice. This suggests that the improvement in the insulin sensitivity triggered by apelin might be secondary to the decrease in adiposity, and it might be due to a direct action in skeletal muscle [90]. 

Myostatin is a myokine of the TGF-β superfamily expressed in both embryonic and adult skeletal muscle that regulates muscle mass and function, producing muscle atrophy [143]. Serum myostatin is upregulated in obesity and correlates with insulin resistance in humans [144]. The overexpression of myostatin in mice causes insulin resistance [145] whereas anti-myostatin antibodies prevent obesity [146] by stimulating fatty acid oxidation and increasing energy expenditure [147]. In addition, it has been reported that myostatin inhibits glucose uptake via the suppression of insulin-dependent and -independent signaling pathways in skeletal muscle [91]. Myostatin inhibits low basal glucose uptake, insulin-induced IRS-1 tyrosine (Tyr495) phosphorylation, and both the expression and activation of PI3K, along with diminishing Akt phosphorylation, which leads to a reduction in insulin-induced GLUT4 membrane translocation and glucose uptake [91]. In addition, this myokine decreases AMPK activity, which is accompanied by reduced GLUT4 gene expression and glucose uptake [91]. Moreover, it has been reported that myostatin regulates glucose metabolism via the AMPK pathway by promoting glucose consumption and glucose uptake, increasing glycolysis, and inhibiting glycogen synthesis in skeletal muscle cells [92]. 

Musclin is a factor secreted by skeletal muscle and is a potent regulator of glucose metabolism [148]. In humans, an increase in the circulating musclin level has been reported in diagnosed T2D patients [149] and those with metabolic syndrome [150]. In the latter, a positive correlation was found between both altered insulinemia and glycemia and a body composition profile with high visceral fat and lean mass [150]. The musclin-induced impairment of insulin-stimulated glucose uptake in skeletal muscle is related to Akt inhibition and PPARγ/LXRα in mice [93] and causes endoplasmic reticulum stress in rats [94]. Recently, it was found that a reduction in muscle-derived musclin production through chronic resistance exercise was involved in improving insulin resistance in rats with T2D [151]. Exercise training improves lipid metabolism and insulin sensitivity by upregulating GLUT4 and downregulating musclin in skeletal muscle [95].

Brain-derived neurotrophic factor (BDNF) is a member of the nerve growth factor family that is generated mainly by the brain [152], but is also secreted by skeletal muscle in response to contractions, and enhances fat oxidation via the activation of the AMP-activated protein kinase [153]. During short-duration aerobic exercise, immediately after a short-duration high-intensity exercise to exhaustion, there is a transient augmentation of serum BDNF concentration in humans [154]. Low levels of BDNF accompanying impaired glucose metabolism in T2D patients have been reported [155]. The repetitive subcutaneous or intracerebroventricular administration of BDNF ameliorates glucose metabolism by enhancing the glucose utilization in muscle in *db/db* mice [96]. Interestingly, a peripheral BDNF treatment promotes GLUT4 protein expression as well as hypophagia in skeletal muscle [97].

## 6. Potential Crosstalk between RONS and Cytokines - Myokines in Glucose Uptake and Insulin Resistance in Skeletal Muscle

It has been reported that the cytokine TNF-α stimulates the production and release of myokines IL-6 and MCP-1 in skeletal muscle. Similarly, an increase in mitochondrial superoxide was observed alongside the activation of redox-sensitive transcription factor NF-κB [156]. This may support the hypothesis that TNF-α induces the release of myokines through the mediation of the increase in ROS, which signals the activation of mediators, such as transcription factors, that are involved in cellular redox signaling pathways. 

Myokines could be relevant in biological functions, including glucose homeostasis, due to the generation of RONS as signaling molecules. During exercise, an increase in the cytosolic ROS production by NOX2 has been reported, which regulates GLUT4 translocation, and the subsequent muscle glucose uptake in skeletal muscle [12]. In addition, insulin elicits an ROS-activated and an IP3-dependent calcium release, both of which impinge on GLUT4 translocation [157]. Therefore, ROS enhances insulin sensitivity, suggesting a pivotal role in glucose uptake [68]. However, there is limited information concerning the role of myokines in RONS production and their effect on glucose transport. As discussed above, irisin plays an important role in glucose metabolism, and this function has been reported via the ROS-mediated AMPK pathway in skeletal muscle cells since the antioxidant N-acetyl cysteine promotes the inhibition of irisin-induced AMPK phosphorylation [82]. To the best of our knowledge, the effect of myokines on the generation of RONS, as well as their impact on GLUT4 translocation and insulin-dependent glucose transport, has not been explored. To date, this field continues to pose unanswered questions that should be addressed in future research.

## 7. Conclusions

RONS are continuously generated in skeletal muscle. A high intracellular level of RONS produces oxidative distress, which compromises cellular viability. However, a moderate level of RONS, called oxidative eustress, is critical and essential to maintain functions and cellular viability in skeletal muscle. 

Hydrogen peroxide and nitric oxide are two specific RONS that are responsible for oxidative eustress, and they act as signaling molecules that interact with components of the cellular signaling pathways that modulate and regulate glucose uptake in skeletal muscle. 

Glucose uptake is crucial for the energy expenditure of the cell since this leads to cellular functions and viability. Furthermore, it is critical in pathophysiological conditions where glucose uptake may be compromised. This is the case in insulin resistance, which emerges in diabetes, obesity, and aging. 

Skeletal muscle releases peptides and proteins, called myokines, into the extracellular space. Myokines may have endocrine and auto/paracrine effects in skeletal muscle and may modulate cellular functions. Some myokines, in addition to other peptides such as cytokines and other factors, may participate in the process of glucose uptake in skeletal muscle. However, the detailed process is still unknown, and therefore, there is a need and demand for further investigations.

Experimental evidence indicates that RONS and some myokines and cytokines participate in the same process, glucose uptake, in skeletal muscle. However, it is unknown how or whether the interplay between these “actors” occurs, and how they might modulate or regulate glucose uptake.

## 8. Future Perspectives

Although the scientific information is limited and scarce, there is great interest in the field to investigate and uncover the details of the process of glucose uptake and how RONS, cytokines and myokines, independently or interacting together, might modulate and regulate this crucial cellular process. This knowledge might be translated to the design of potential pharmacological or functional therapies for the treatment of insulin resistance in diabetes, obesity, and aging.

It must be considered that the study of RONS is particularly difficult due to the inherent properties of these molecules, since they present high reactivity with other molecules, produce nonspecific reactions, and have a short half-life. This complicates the research undertaken in pathophysiological models of skeletal muscle, in which the effect of cytokines and myokines may be investigated in the context of glucose uptake in skeletal muscle. Although the research depends on the available resources and the equipment, training and imagination of scientists to develop proper models to adapt to this kind of work, we propose that studies use the model of isolated skeletal muscle fibers [28,29,30,36,42,67,158,159,160,161] to analyze the expression of genetically encoded biosensors for the specific detection of RONS in these fibers, in combination with fluorescence microscopy imaging quantitative analysis [162,163]. These methodological approaches permit the spatiotemporal monitoring of RONS activity and may help evaluate the effect of cytokines and myokines in glucose uptake in these cells. Moreover, the studies may be complemented with molecular biology techniques.

Finally, the aim of this review has been to highlight that RONS, cytokines and myokines, either independently or interplaying, may modulate and regulate glucose uptake in skeletal muscle. And this might be translated to the future design of potential therapies for the treatment of insulin resistance in pathologies, such as diabetes, obesity, and aging. Since the current knowledge is limited, we encourage colleagues and researchers in the field to lead future investigations to uncover the process of glucose uptake in skeletal muscle.

## Figures and Tables

**Table 1:** Main effects of cytokines and myokines on insulin sensitivity in skeletal muscle.

Peptide	Type	Main Effects	References
**Adiponectin**	Adipokine	↑ Glucose uptake. ↑ GLUT4 translocation via AMPK.	[71,72] [73]
**Leptin**	Adipokine	↑ Glucose uptake. ↑ GLUT4 translocation independent of AMPK. ↓ Expression and activity of the negative regulators of AS160.	[74] [75] [75]
**Monocyte chemotactic protein 1**	Inflammatory cytokines	↑ Recruitment of macrophages and altered local insulin sensitivity. ↓ Insulin signaling and glucose uptake.	[76] [77]
**Interleukin-1β**	Inflammatory cytokines and myokine	↓ GLUT4 translocation, glucose uptake in response to insulin and the expression of IRS-1.	[67,78]
**Tumor necrosis factor-** **α**	Inflammatory cytokines	¬ AS160 phosphorylation. ↑ Serine phosphorylation at insulin receptor and IRS-1.	[79] [80]
**Fibroblast growth factor 21**	Hepatokine and myokine	↑ Glucose uptake and GLUT4 translocation dependent on PKC-ζ.	[81]
**Irisin**	Myokine	↑ Glucose uptake via ROS-mediated AMPK pathway. Reverses insulin resistance via p38-MAPK-PGC-1α. ↑ Fatty acid oxidation and glucose utilization by AMPK.	[82,83] [84] [85]
**Interleukin-6**	Myokine	↑ Insulin sensitivity via AMP-activated protein kinase. ↑ Glucose metabolism while resting, without changing insulin-stimulated glucose transport and insulin signaling.	[86] [87]
**Apelin**	Adipokine and myokine	↑ Glucose uptake via endothelial NO synthase, AMPK and Akt.	[88,89,90]
**Myostatin**	Myokine	¬ Glucose uptake via suppression of insulin-dependent and -independent signaling pathways in skeletal muscle. Acceleration of glucose utilization via AMPK. Up-regulation of several glucose metabolism-related genes.	[91] [92]
**Musclin**	Myokine	↓ Insulin-stimulated glucose uptake via Akt inhibition and PPARγ/LXRα. ↓ Glucose metabolism and, at least in part, through endoplasmic reticulum stress. Exercise training improves ↑ insulin sensitivity by upregulating GLUT4 and downregulating musclin in skeletal muscle.	[93] [94] [95]
**Brain-derived neurotrophic factor**	Myokine	↑ Glucose uptake. ↑ GLUT4 protein expression.	[96] [97]

↓: decrease; ↑: increase; ¬: inhibit.
